# Protein Interactions from Complexes: A Structural Perspective

**DOI:** 10.1155/2007/49356

**Published:** 2006-12-20

**Authors:** Luke Hakes, David L. Robertson, Stephen G. Oliver, Simon C. Lovell

**Affiliations:** Centre for the Analysis of Biological Complexity, Faculty of Life Sciences, The University of Manchester, Michael Smith Building, Oxford Road, Manchester M13 9PT, UK

## Abstract

By combining crystallographic information with protein-interaction data obtained through traditional experimental means, this paper determines the most appropriate method for generating protein-interaction networks that incorporate data derived from protein complexes. We propose that a combined method should be considered; in which complexes composed of five chains or less are decomposed using the matrix model, whereas the spoke model is used to derive pairwise interactions for those with six chains or more. The results presented here should improve the accuracy and relevance of studies investigating the topology of protein-interaction networks.

## 1. INTRODUCTION

The working of living cells is underpinned by an almost overwhelming array of molecular interactions that form a complex and multifaceted network: “the interactome.” The explosive growth in both the type and volume of experimental data available to researchers interested in elucidating the properties of the interactome has led to a wide range of studies investigating a number of the core biological networks. Networks of gene regulation [1], metabolism [2–4], and protein interactions [5] have all been studied using data from the yeast *Saccharomyces cerevisiae*. The majority of cellular processes are mediated by protein-protein interactions, including signal transduction pathways and the regulation of gene expression. Thus protein-interaction networks have been afforded the most attention, in the hope that knowledge of their structure and topology will help us to understand their functions and evolutionary history.

Recently, there have been a number of large-scale protein-interaction studies. Efforts have ranged from studies that investigated only a subset of the protein interactome to attempts identifying all protein interactions within the cell [6]. It is clear that none of these studies, when taken individually, constitute a comprehensive picture of the underlying biology. Therefore, in order to derive the greatest benefit from their analyses, researchers wishing to undertake computational studies of the protein-interaction network as a whole must first integrate the results of many individual experiments in order to produce a single unified network representation. Typically, this is done by use of a graph-theoretical approach in which the proteins within the network are depicted as nodes, with interacting proteins connected by undirected links (edges) [7].

Generating this type of network representation is relatively easy for studies employing technologies that identify interactions in a binary fashion, such as the yeast two-hybrid system Y2H [8]. However, for experiments that identify all the proteins within a given complex, the process of defining a set of pairwise protein-protein interactions is more difficult. Protein complexes are often isolated in an affinity-purification experiment in which a single protein (the bait) is provided with a molecular tag (such as FLAG [9] or TAP [10]) that allows the purification of the “bait” together with all of the “prey” proteins that belong to the same multiprotein complex. It is unlikely that every “prey” protein interacts directly with the “bait” protein used to purify the complex; rather, the topology of the complex will include both “bait-prey” and “prey-prey” interactions. The true topology of these experimentally derived complexes cannot be determined from the individual experiments themselves, although some progress towards achieving this goal has been made by combining different datasets [11].

**Figure 1:**
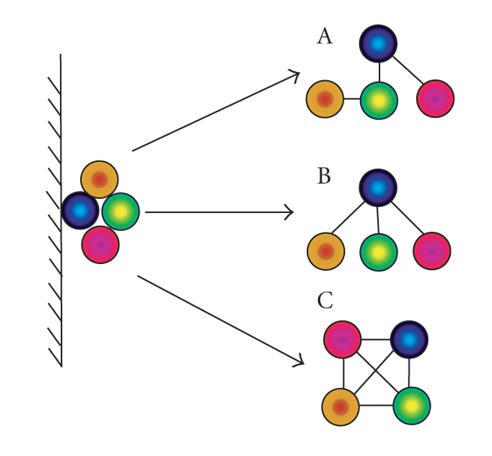
*Possible modeling methodologies for experimentally determined protein complexes:* (A) actual topology of protein complex; (B) spoke model, interactions are assigned between bait (blue) and each captured prey; (C) matrix model, all possible interacting pairs are assumed. Balls represent polypeptide chains within a protein complex; lines between balls represent a physical interaction between those chains.

In practice, a model is used, in which pairwise interactions are assigned by applying either a matrix- or spoke-based modelling methodology to each complex [12, 13] (Figure 1). Recently, we showed that the choice of modelling methodology has a dramatic effect on the topological features of the protein-interaction network [14]. However, it is unclear, at present, which of the two proposed models (if either) should be selected when performing this type of analysis. In this study, we draw on crystallographic data within the protein quaternary structure (PQS) database [15] to determine the actual topology of the protein complexes under investigation.

## 2. METHODS

**Figure 2:**
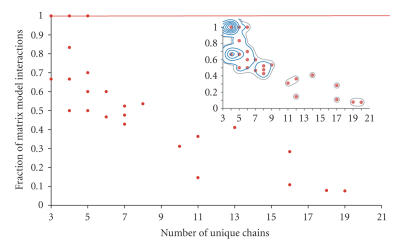
*Performance of the matrix model on 133 structures with different numbers of unique polypeptide chains.* The matrix model performs well for structures ≤ 5 chains, illustrated by the large number of complexes in this region of the graph that are either fully connected (illustrated by the red line) or have large numbers of connections between member chains. Inset: density plot showing complex size distribution, ≈ 50% of all complexes have ≤ 5 unique chains. Tighter contours represent increasing numbers of protein chains.

We began by extracting all available protein complexes from the PQS database (≈ 32,000 structures as of 29/07/05). An automated protocol was then used to triage the collection until a set of complexes that fulfilled the criterion for analysis remained. That is, they were at least heterotrimeric in nature (i.e., they contained at least three polypeptide chains, with unique amino-acid sequences of greater than or equal to 30 residues), as analysis of the connectivity of complexes with less than three unique chains is clearly meaningless. This resulted in approximately 900 protein complexes that were then further filtered to identify and remove redundancy. Subcomplexes were collapsed into their parents and redundant structures were identified by performing an all-against-all comparison of each protein complex. Those complexes sharing two thirds or more of their chains with others in the set were then removed. The 133 protein complexes that remained after this triage procedure were then subjected to further analysis. Actual physical interactions between polypeptide chains within the complexes were identified by computational analysis of the crystal structure, using an empirically equivalent algorithm to the full atom contact (FAC) method employed by Gong et al. [16], with an interaction between two chains defined as the occurrence of at least five instances in which C*α* atoms within the chains come within 7.5 Å of each other.The ability of the matrix model to define the real set of interactions within these complexes was then assessed by plotting the actual number of interactions in a complex (as a percentage of those defined by the matrix model) against the number of unique chains within that complex (Figure 2).

We then performed a direct comparison between the matrix and spoke models by combining crystallographic data with protein complex data obtained experimentally for the yeast *Saccharomyces cerevisiae* [9, 10, 17, 18]. First, we further triaged our protein complex dataset to include only (at least) heterotrimeric complexes composed of unique chains that had experimentally determined homologs in yeast. By extracting the protein sequence of each chain from every complex within the PQS and blasting it against the yeast genome (cutoff; *e* ≦ 1*e* ^−10^, fraction of conserved residues ≧ 35), we were able to identify, and make use of, protein complexes determined in other species that are structural homologs of those found in *Saccharomyces cerevisiae*. After collapsing subcomplexes into their parents and removing any redundant structures, we were left with a set of thirteen structures that were suitable for use in the final part of our analysis. Crystallographic data for each structure and the FAC method were used to determine the “true topology” of the interactions within a complex and the ability of each model to describe it was assessed by calculating a score based on the following functions:

(1) Smat = TP − FP;

(2) Sspo = TP − FN − FP,

where, for the matrix model (1), TP is the number of interactions observed in both the crystal structure and in the experimental network and FP (3) is the number of false positive interactions between the polypeptide chains, calculated using the formula

(3) $$\text{FP}=\frac{N^{2}-N}{2}-\text{TP}$$,

where *N* is the number of unique polypeptide chains in the crystal structure. For the spoke model (2), TP is as above, FN is the false-negative count, calculated by considering interactions between proteins that are identified in the crystal structure and that were used as baits in high-throughput protein-interaction studies, but for which no experimental interaction was identified. Finally, FP is the false-positive count; the number of interactions identified between proteins in an experimentally determined complex that do not occur in the actual crystal structure. All self-interactions identified in both the experimental and PQS complexes were excluded.

## 3. RESULTS AND DISCUSSION

Analysis of protein connectivity within complexes over a range of sizes revealed that those complexes composed of ≤ 5 unique polypeptide chains are generally appropriately described by the matrix model (as can be seen from the points lying along the red line of the plot in Figure 2). However, as the number of chains within a complex increases, its topology is less and less likely to be described by the fully connected graph specified by the matrix model and the “performance” of this model rapidly diminishes. This suggests that for complexes composed of > 5 chains, the application of the spoke model is a more appropriate choice.

In order to test this hypothesis, we performed a direct comparison of the two models by applying our scoring functions to the eleven usable structures that we had identified as either being purified from yeast or that we could say had an identifiable yeast homolog. As expected, this analysis revealed that (in every case) the matrix model performed as well or better than the spoke model for complexes containing up to five unique chains (Table 1). The superior performance of the spoke model and the expected reduction in performance of the matrix model for complexes with higher chain numbers were also observed, supporting the hypothesis that the spoke model provides the appropriate description of these larger complexes. Therefore, we suggest that when using this type of data to construct protein-interaction networks, the optimal method for decomposing the interactions into node-edge relationships is a combined one, with the matrix model used for complexes of five chains or less, and the spoke model for complexes of six chains or more.

Clearly, no single model (either matrix or spoke) can provide the true representation of the actual interactions that occur within protein complexes and we make no attempt here to state that this is the case. Rather, we aim to suggest a method by which experimental data from studies elucidating protein complexes are best processed so that they most accurately depict reality, prior to their incorporation into a global protein-interaction network and its subsequent analysis using graph-theoretical methods.

It has long been assumed that the matrix model provides a relatively poor description of the true interaction space for any given complex, and previous work by Bader et al. demonstrated that the spoke model was more accurate (in agreement with published literature) [12]. The present study is complementary to, and an extension of, earlier work that aimed at validating experimentally determined interactions [19]. Edwards et al. compared the topology of three large protein complexes of known structure to a wide range of proteomics data, in order to estimate the error rate associated with the matrix model. We have taken this analysis a step further, and in addition to assessing the performance of the matrix model using a larger dataset of known structures (133), we also assess the performance of the spoke model and suggest which is likely to have the better performance and in what circumstances. We find that the large complexes studied by Edwards et al. [19] are more appropriately described by the spoke model, suggesting that their estimates of error rates may be pessimistic. This improvement in our understanding of how the individual proteins within complexes interact, and the increase in clarity about how data on protein complexes derived from proteomics studies are best processed should allow us to produce more accurate and meaningful network representations.

Crystal structures of large complexes provide the best way of validating the protein-protein interaction networks and for developing appropriate models for integrating and interpreting data from high-throughput studies that employ techniques like Y2H and TAP-tagging. Although, in general, the solution of protein crystal structures is becoming more automated, the structures of protein complexes must still be solved by careful and painstaking validation of the crystallized complex at each stage. Structural genomic initiatives will often systematically miss these complexes because they generally attempt only to produce, crystallize, and solve the structures of individual proteins. In this light, the lack of overlap between structural data and network interaction data, while striking, is not unexpected.

It should be noted that our analysis methodology regards the structural data deposited within the PQS database as being representative of the “real” biological unit (BU). In reality, the data provided by PQS is a prediction of the BU based on the crystallographic asymmetric unit (ASU). However, as these predictions have been found to be accurate in approximately 75% of tested cases [20], it is unlikely to substantially affect the general trends observed here.

While the paucity of overlap between the structurally solved protein complexes and those determined experimentally precluded any type of rigorous statistical analysis, we believe, given the result shown in Figure 2 (which covers 133 unique protein structures) and the clear trend identified in the direct comparison of the models, that our conclusions are valid.

## Figures and Tables

**Table 1:** *Scores for each of the PQS structures that passed the filtering criterion.* STP is spoke-model true positives, SFP is spoke-model true negatives, SFN is spoke-model false negatives, MTP is matrix-model true positives, MFP is matrix-model false positives, MFN is matrix-model false negatives. Score spoke is overall score for spoke model, score matrix is overall score for matrix model. Bold scores indicate best performing model, underlining is used when both models perform equally well.

Structure	Description	Chains	STP	SFP	SFN	MTP	MFP	MFN	Score spoke	Score matrix

1iru	20S proteasome	12	26	40	0	26	40	0	*−14*	*−14*
1k8a	Large ribosomal subunit	12	0	0	3	5	61	0	−3	−56
1y1v	RNA polymerase II-TFIIs	12	15	32	3	18	48	0	−20	−30
1n32	Small ribosomal subunit	9	1	14	1	3	33	0	−14	−30
1sxj	RFC bound to PCNA	5	6	4	0	6	4	0	*2*	*2*
1u2v	ARP2/3	5	4	2	1	6	4	0	1	**2**
1id3	Nucleasome	4	2	0	3	5	1	0	−1	**4**
1gw5	AP2	3	3	0	0	3	0	0	*3*	*3*
1kyo	Cytochrome BC1	3	1	0	1	2	1	0	0	**1**
1ntk	Cytochrome BC1	3	0	0	1	1	1	0	−1	−1
1qo1	ATP synthase motor	3	1	0	2	3	0	0	−1	**3**

## References

[1] Amoutzias GD, Robertson DL, Oliver SG, Bornberg-Bauer E (2004). Convergent evolution of gene networks by single-gene duplications in higher eukaryotes. *EMBO Reports*.

[2] Jeong H, Tombor B, Albert R, Oltvai ZN, Barabási A-L (2000). The large-scale organization of metabolic networks. *Nature*.

[3] Ma H, Zeng A-P (2003). Reconstruction of metabolic networks from genome data and analysis of their global structure for various organisms. *Bioinformatics*.

[4] Wagner A, Fell DA (2001). The small world inside large metabolic networks. *Proceedings of the Royal Society B: Biological Sciences*.

[5] Wuchty S (2004). Evolution and topology in the yeast protein interaction network. *Genome Research*.

[6] Ito T, Chiba T, Ozawa R, Yoshida M, Hattori M, Sakaki Y (2001). A comprehensive two-hybrid analysis to explore the yeast protein interactome. *Proceedings of the National Academy of Sciences of the United States of America*.

[7] Barabási A-L, Oltvai ZN (2004). Network biology: understanding the cell's functional organization. *Nature Reviews Genetics*.

[8] Fields S, Song O-K (1989). A novel genetic system to detect protein-protein interactions. *Nature*.

[9] Ho Y, Gruhler A, Heilbut A (2002). Systematic identification of protein complexes in Saccharomyces cerevisiae by mass spectrometry. *Nature*.

[10] Gavin A-C, Bösche M, Krause R (2002). Functional organization of the yeast proteome by systematic analysis of protein complexes. *Nature*.

[11] Scholtens D, Vidal M, Gentleman R (2005). Local modeling of global interactome networks. *Bioinformatics*.

[12] Bader GD, Hogue CWV (2002). Analyzing yeast protein-protein interaction data obtained from different sources. *Nature Biotechnology*.

[13] von Mering C, Krause R, Snel B (2002). Comparative assessment of large-scale data sets of protein-protein interactions. *Nature*.

[14] Hakes L, Robertson DL, Oliver SG (2005). Effect of dataset selection on the topological interpretation of protein interaction networks. *BMC Genomics*.

[15] Henrick K, Thornton JM (1998). PQS: a protein quaternary structure file server. *Trends in Biochemical Sciences*.

[16] Gong S, Yoon G, Jang I (2005). PSIbase: a database of protein structural interactome map (PSIMAP). *Bioinformatics*.

[17] Gavin A-C, Aloy P, Grandi P (2006). Proteome survey reveals modularity of the yeast cell machinery. *Nature*.

[18] Krogan NJ, Cagney G, Yu H (2006). Global landscape of protein complexes in the yeast Saccharomyces cerevisiae. *Nature*.

[19] Edwards AM, Kus B, Jansen R, Greenbaum D, Greenblatt J, Gerstein M (2002). Bridging structural biology and genomics: assessing protein interaction data with known complexes. *Trends in Genetics*.

[20] McMullan D, Canaves JM, Quijano K (2005). High-throughput protein production for X-ray crystallography and use of size exclusion chromatography to validate or refute computational biological unit predictions. *Journal of Structural and Functional Genomics*.

